# Ubiquitination and Degradation of Ribonucleotide Reductase M1 by the Polycomb Group Proteins RNF2 and Bmi1 and Cellular Response to Gemcitabine

**DOI:** 10.1371/journal.pone.0091186

**Published:** 2014-03-10

**Authors:** Yingtao Zhang, Xin Li, Zhengming Chen, Gerold Bepler

**Affiliations:** Molecular Therapeutics Program, Karmanos Cancer Institute, Detroit, Michigan, United States of America; Academia Sinica, Taiwan

## Abstract

Ribonucleotide reductase M1 (RRM1) is required for mammalian deoxyribonucleotide (dNTP) metabolism. It is the primary target of the antimetabolite drug gemcitabine, which is among the most efficacious and most widely used cancer therapeutics. Gemcitabine directly binds to RRM1 and irreversibly inactivates ribonucleotide reductase. Intra-tumoral RRM1 levels are predictive of gemcitabine’s therapeutic efficacy. The mechanisms that regulate intracellular RRM1 levels are largely unknown. Here, we identified the E3 ubiquitin-protein ligases RNF2 and Bmi1 to associate with RRM1 with subsequent poly-ubiquitination at either position 48 or 63 of ubiquitin. The lysine residues 224 and 548 of RRM1 were identified as major ubiquitination sites. We show that ubiquitinated RRM1 undergoes proteasome-mediated degradation and that targeted post-transcriptional silencing of RNF2 and Bmi1 results in increased RRM1 levels and resistance to gemcitabine. Immunohistochemical analyses of 187 early-stage lung cancer tumor specimens revealed a statistically significant co-expression of RRM1 and Bmi1. We were unable to identify suitable reagents for *in situ* quantification of RNF2. Our findings suggest that Bmi1 and possibly RNF2 may be attractive biomarkers of gemcitabine resistance in the context of RRM1 expression. They also provide novel information for the rational design of gemcitabine-proteasome inhibitor combination therapies, which so far have been unsuccessful if given to patients without taking the molecular context into account.

## Introduction

The antimetabolite gemcitabine (2′, 2′-difluoro-2′-deoxycytidine) is one of the principal agents used for treatment of malignancies. Ribonucleotide reductase M1 (RRM1) is the regulatory subunit of ribonucleotide reductase and required for deoxyribonucleotide (dNTP) synthesis. Gemcitabine diphosphate binds to RRM1 and irreversibly inactivates ribonucleotide reductase [1], [2], [3]. RRM1 is also involved in cell proliferation, migration, and invasion [4], [5].

Although several molecules involved in dNTP metabolism have been reported to be predictive of cellular response to gemcitabine, only RRM1 has been validated in independent studies [6], [7], [8], [9], [10]. High levels cause gemcitabine resistance and low levels sensitivity. Mechanisms that control RRM1’s abundance are largely unknown, but may provide an opportunity for optimization of gemcitabine efficacy. Using a variety of screening methods for RRM1-associated proteins including yeast two-hybrid screening, we identified the RING domain-containing E3 ubiquitin-protein ligases RNF2 (RING finger protein 2) and Bmi1 (B cell-specific moloney murine leukemia virus insertion site 1). RNF2 and Bmi1 belong to the polycomb group protein (PcG) family, and both are involved in the maintenance of histone H2A levels through ubiquitination and the E3 ubiquitin ligase complex through transcriptional repression [11], [12].

Ubiquitination commonly targets proteins for degradation, but it is also involved in protein transport and DNA damage repair among others [13]. It is a multistep process that results in the covalent, reversible linkage of ubiquitin to lysine residues of the target. Substrate specificity is determined by E3 ubiquitin-protein ligases [14], [15]. RING domain genes, which include the polycomb-repressive complex-1 genes RNF2 and Bmi1, are one of several classes of these ligases [16].

We describe the RRM1, RNF2, and Bmi1 interaction and co-localization, the mechanisms by which RNF2 and Bmi1 regulate RRM1 levels and cellular response to gemcitabine, and *in situ* protein levels in tumor specimens derived from patients with lung cancer. Potential therapeutic applications are discussed.

## Results

### RNF2 and Bmi1 are Associated with RRM1

**Figure 1:**
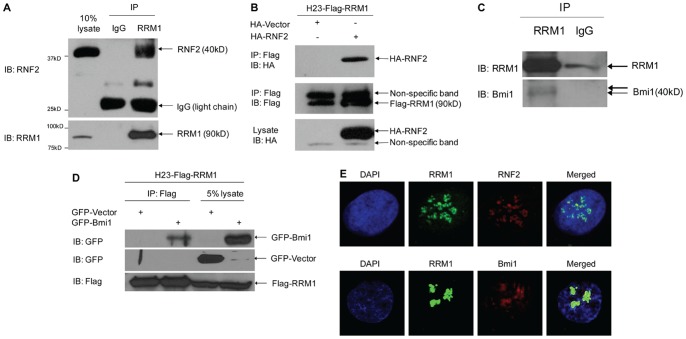
RRM1 interacts with RNF2 and Bmi1. A) Endogenous interaction between RRM1 and RNF2 in H23-WT. Cell lysate was subjected to IP using control IgG Ab (lane 2) or anti-RRM1 Ab (lane 3), followed by immunoblotting (IB) with the indicated antibodies. Expression of RNF2 and RRM1 in cell lysates (lane 1). B) Flag-RRM1 interacts with HA-RNF2 in H23-R1 (stably expressing Flag-RRM1 in H23-WT). H23-Flag-RRM1 was transfected with HA-RNF2. IP was performed with anti-Flag Ab, followed by IB with the indicated antibodies. C) Endogenous interaction between RRM1 and Bmi1 in H23 gemcitabine-resistant (GR) cells. IP was performed with anti-RRM1 Ab, followed by IB with the indicated antibodies. D) Flag-RRM1 interacts with GFP-Bmi1 in H23-Flag-RRM1. H23-Flag-RRM1 was transfected with GFP-Bmi1. IP was performed with anti-Flag Ab, followed by IB with the indicated antibodies (lanes 1&2). IB of cell lysates (lanes 3&4). E) Co-localization of RRM1 with RNF2 and Bmi1 in H23-WT. Confocal immunofluorescence microscopy was carried out by co-staining cells with anti-RRM1 (green) and anti-RNF2 or anti-Bmi1 (red) Abs. Nuclei were counterstained with DAPI (blue).

We identified RNF2 as an RRM1-interacting protein using yeast two-hybrid screening (data not shown). To determine whether RNF2 interacts with RRM1 in mammalian cells, the adenocarcinoma of the lung derived cell line NCI-H23 (H23-WT) was subjected to immunoprecipitation (IP). As shown in Fig. 1A, RNF2 was co-precipitated by an anti-RRM1 antibody (Ab) but not the control IgG Ab, indicating that an intracellular interaction between RNF2 and RRM1 exists. To test whether transfected RNF2 and RRM1 proteins interact, a stably expressing Flag-labeled RRM1 cell line (H23-Flag-RRM1) was transiently transfected with hemagglutinin-(HA)-labeled RNF2. HA-RNF2 was detected by an anti-HA Ab using IP with an anti-Flag Ab (Fig. 1B, top panel, Flag-RRM1 probed with anti-Flag Ab is shown in the middle panel and HA-RNF2 probed with anti-HA Ab in the bottom panel). To investigate the endogenous interaction between RRM1 and Bmi1, H23 gemcitabine-resistant (GR) cells were subjected to IP. We detected a faint Bmi1 band after RRM1 pull-down (Fig. 1C). Under low-stringency conditions, some RRM1 binds unspecifically to control beads (Fig. 1C). We then transiently transfected H23-Flag-RRM1 with GFP-Bmi1 and found that GFP-Bmi1 also interacts with Flag-RRM1 (Fig. 1D, GFP-Bmi1 transfection IP and lysate probed with an anti-GFP Ab in the top panel, GFP-vector control in the middle panel, anti-Flag Ab in the bottom panel). Using confocal microscopy with immunofluorescence staining, we were able to confirm nuclear co-localization of endogenous RNF2 or Bmi1 with RRM1 in H23-WT (Fig. 1E).

### RRM1 Undergoes Proteasome-mediated Degradation

**Figure 2:**
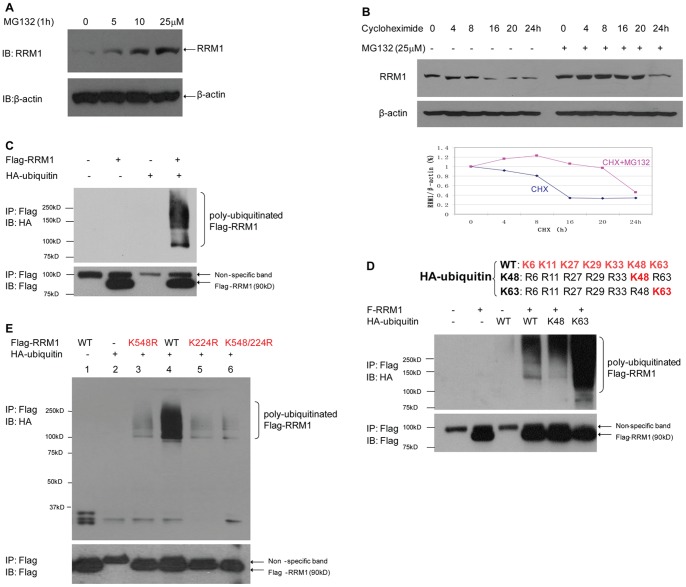
RRM1 is Poly-Ubiquitinated and Degraded by Proteasomes. A) RRM1 accumulates in the presence of the proteasome inhibitor MG132 in H23-WT. Cells were treated at the indicated concentrations for 1 h (lanes 2, 3&4). IBs of total cell lysates show a dose-dependent RRM1 accumulation (top panel). β-actin was used as loading controls (bottom panel). B) RRM1 levels decline in the presence of 100 µg/ml cycloheximide (lanes 1–6, top panel), MG132 delays the decline by at least 8 h in H23-WT (lanes 8–13, top panel). β-actin was used as loading controls (bottom panel). RRM1 signals normalized by β-actin signals are plotted in the graph. C) RRM1 is poly-ubiquitinated. AD293 was co-transfected with Flag-RRM1 and HA-ubiquitin. After 48 h, IP was performed using anti-Flag Ab under denaturing conditions, followed by IB with the indicated antibodies. D) RRM1 poly-ubiquitination via K48 and K63 linkage in AD293. Flag-RRM1 and HA-ubiquitin wild type (WT) and mutants K48 (only one lysine at 48 position) and K63 (only one lysine at 63 position) were co-transfected. After 48 h, IP was performed using anti-Flag Ab under denaturing conditions, followed by IB with the indicated antibodies. E) Lysine 548 and 224 are major ubiquitination sites of RRM1. AD293 was transfected with wild-type RRM1 or RRM1 mutants (K548R, K224R, and K548/224R) together with HA-ubiquitin. After 48 h, IP was performed using anti-Flag Ab under denaturing conditions, followed by IB with the indicated antibodies.

Next, we examined if RRM1 is degraded by the ubiquitin–proteasome pathway. H23-WT cells were treated with the proteasome inhibitor MG132. RRM1 protein levels accumulated in its presence in a dose-dependent manner (Fig. 2A). To test whether the increase in RRM1 levels by MG132 was due to decreased protein degradation, cells were treated with 100 µg/ml cycloheximide to block protein synthesis in serum free medium, and RRM1 levels decreased dramatically (Fig. 2B, lanes 1–6). The addition of 25 µM MG132 resulted in RRM1 protein accumulation (Fig. 2B, lanes 8–13), suggesting that RRM1 expression is regulated by proteasome mediated degradation.

To determine if RRM1 is ubiquitinated, we co-expressed Flag-labeled RRM1 with HA-labeled ubiquitin in adenovirus transformed human embryonic kidney cells (AD293). IP with an anti-Flag Ab and probed with anti-HA revealed poly-ubiquitinated RRM1 protein bands (Fig. 2C). Since ubiquitin chains can be linked via lysine at either position 48 or 63 of ubiquitin, the former being a mark for proteasome degradation and the latter for other cellular functions such as protein transport and DNA damage repair, we examined RRM1’s poly-ubiquitination in AD293 cells. Flag-labeled RRM1 was co-transfected with either wild-type or mutant ubiquitin. The mutant ubiquitin had only one lysine residue, either at position 48 (K48) or at 63 (K63). We were able to detect protein bands corresponding to poly-ubiquitinated RRM1 with both mutants (Fig. 2D), indicating that RRM1 is not only a substrate of ubiquitin for proteasome-mediated degradation but also for other cellular functions.

We then identify the ubiquitin attachment sites on RRM1. Using ubiquitination prediction software UbiPred (http://iclab.life.nctu.edu.tw/ubipred), UbPred (http://ubpred.org/), and BDM-PUB (http://bdmpub.biocuckoo.org/), several potential sites in both the N- and C-terminal region were found, including Lys 548 and Lys 224 with scores of 0.83 and 0.50, respectively. We generated the RRM1 mutants K548R, K224R, and K548/224R by substitution of lysine with arginine. As showed in Fig. 2E, a substantial ubiquitination reduction was observed with all three mutants; however, even K548/224R did not completely abolish RRM1 ubiquitination, suggesting other lysine residues may also serve as ubiquitin attachment sites.

### RNF2 and Bmi1 Induce Poly-ubiquitination of RRM1 as E3 Ubiquitin Ligases

**Figure 3:**
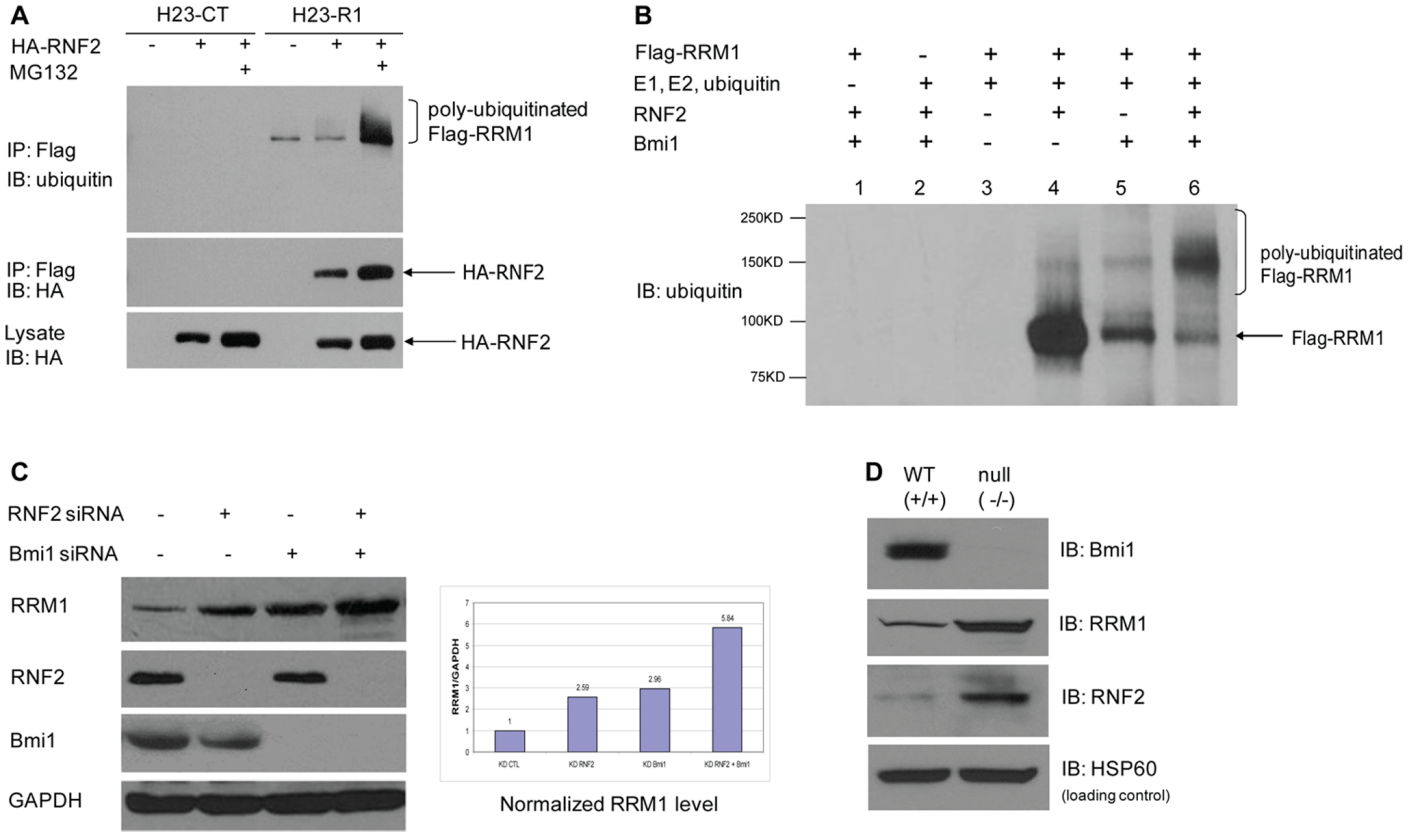
RNF2 and Bmi1 Induce Poly-Ubiquitination of RRM1. A) RNF2 induces poly-ubiquitination of RRM1. H23-Flag-RRM1 and H23-Flag-Control cells were transfected with HA-RNF2 or HA-Vector. After 48 h, cells were treated with MG132. IP was performed using anti-Flag Ab followed by IB with the indicated antibodies (top and middle panel). The bottom panel shows expression of HA-RNF2 in cell lysates. B) *In vitro* ubiquitination assay showing that RNF2 and Bmi1 are E3 ligases for RRM1 ubiquitination. RRM1 protein was incubated with RsNF2 (lane 4) and Bmi1 (lane 5) or both together (lane 6) along with E1, UbcH5b and UbcH5c (E2), ubiquitin, and an energy-regenerating system. C) Depletion of RNF2 and Bmi1 by siRNA increases RRM1 levels. siRNA to RNF2 and Bmi1 or control siRNA were transfected to H23-WT. IP was performed using the indicated antibodies. RRM1 signals normalized by GAPDH signals are plotted in the graph. D) RRM1 accumulates in Bmi1 null MEFs. Bmi1 wild type and null MEFs were lysed and analyzed by Western blotting using the indicated antibodies. HSP60 was used as loading control (bottom panel).

We tested if RRM1 is an E3 substrate of RNF2 and Bmi1 by using *in vivo* and *in vitro* ubiquitination assays. H23-Flag-RRM1 and H23 vector-transfected (H23-Ct) cells, both carrying a Flag-label, were transiently transfected with HA-RNF2 or HA-vector and then treated with MG132. IP with anti-Flag Ab showed when RNF2 was coexpressed with RRM1 and treated with the proteasome inhibitor MG132, higher molecular weight ladders were detected (Fig. 3A, top panel), indicating that RNF2 is an E3 ubiquitin ligase of RRM1. In addition, HA-labeled RNF2 levels were only detectable in H23-Flag-RRM1 cells, and they were higher with MG132 treatment (Fig. 3A, middle panel). The bottom panel of Fig. 3A shows RNF2 expression in the same whole cell lysates using immunoblotting with anti-HA Ab.

Next, we tested the ability of RNF2 and Bmi1 to catalyze RRM1 ubiquitination *in vitro*. RNF2 and Bmi1 stimulated the poly-ubiquitination of RRM1 in the presence of purified E1, ubiquitin-conjugating enzyme (E2), and ubiquitin (Fig. 3B). These data also suggest that RNF2 and Bmi1 together are more efficient in generating RRM1 poly-ubiquitination than either alone.

Since E3 ligases promote substrate degradation, we investigated if RRM1 levels increase when RNF2 and Bmi1 are silenced. We indeed found that depletion of endogenous RNF2 and Bmi1 by siRNA, alone or together, increased RRM1 protein levels approximately 5-fold in H23-WT cells (Fig. 3C). Finally, we observed that expression of endogenous RRM1 is increased in Bmi1 null mouse embryonic fibroblasts (MEFs; Fig. 3D). Surprisingly, RNF2 levels were also increased (Fig. 3D). This may be explained by a reduced self-ubiquitination activity of RNF2 [17], which is promoted by Bmi1 [18] thus leading to RNF2 accumulation. In contrast, RNF2 levels were not significantly altered after partial depletion of Bmi1 using siRNA (Fig. 3 C). Ubiquitination of histone H2A is not changed after Bmi1 knockout, probably because of the compensatory increase of RNF2.

### RNF2 and Bmi1 are Involved in Cellular Response to Gemcitabine

**Figure 4:**
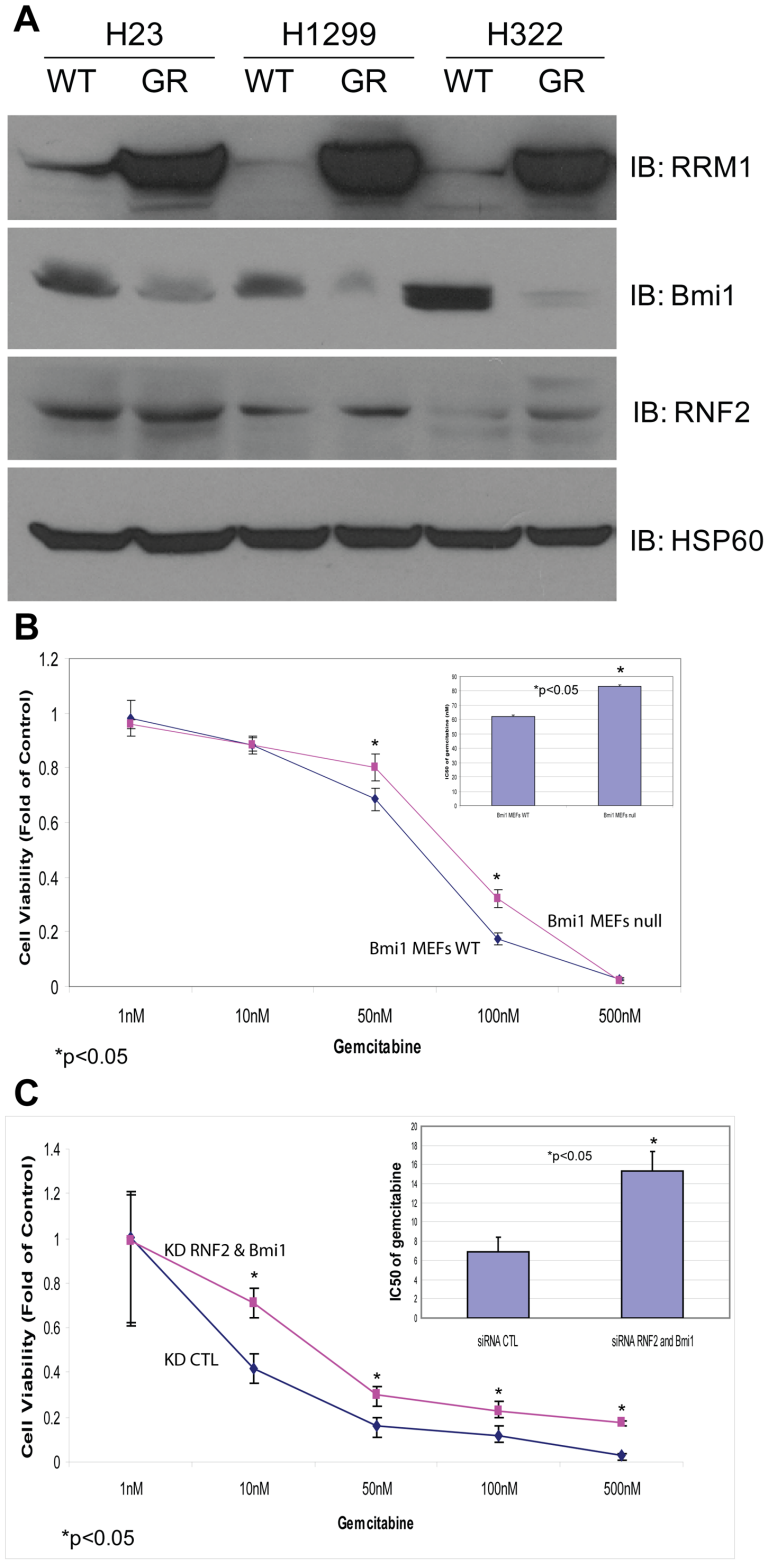
RNF2 and Bmi1 Modulate Cellular Response to Gemcitabine. A) Gemcitabine-resistant (GR) cell lines display upregulation of Bmi1 protein. The GR cell lines H23, H322, and H1299 were analyzed by Western blotting using the indicated antibodies. HSP60 was used as loading control (bottom panel). B) *In vitro* drug sensitivity assay of Bmi1 null MEFs indicating increased resistant (*p*<0.05) to gemcitabine. All experiments were conducted in triplicate and error bars represent the standard deviation (s.d.). C) Co-depletion of RNF2 and Bmi1 by siRNA induces gemcitabine resistance. H23-WT was transfected with control or RNF2 and Bmi1 siRNA and treated with gemcitabine at the indicated concentrations. Error bars represent means ± s.d. of three independent experiments. There was a 2.2-fold increase in the gemcitabine IC50 with silencing of RNF2 and Bmi1 compared to controls (*p*<0.05).

Since RRM1 expression levels determine the therapeutic efficacy of gemcitabine and RNF2 and Bmi1 regulate RRM1 protein levels, we investigated if RNF2 and Bmi1 regulate the cellular response to gemcitabine. First, we determined Bmi1 levels in wild-type and gemcitabine-resistant (GR) clones of the NSCLC cell lines H23, H322, and H1299. Gemcitabine resistance was generated by treating WT cells with 1 nM gemcitabine for two weeks. Surviving cells were then exposed to increasing doses until cells survived drug concentrations ten-fold higher than the original gemcitabine IC50. Clonal sublines of resistant cells were generated from single colonies, and resistance was confirmed using a cell viability assay. As expected, we found dramatic upregulation of RRM1 levels in GR compared to WT cells (Fig. 4A). Bmi1 levels were dramatically reduced in all three GR cells (Fig. 4A). However, RNF2 levels showed little change (Fig. 4A), probably for the same reason as discussed above for siRNA induced partial Bmi1 reduction. This suggests that Bmi1 rather than RNF2 levels are inversely associated with RRM1 levels in GR cells.

The IC50 for gemcitabine was 83.1 nm in Bmi1 null cells compared to 62.1 nM for Bmi1 WT MEFs, which was significantly higher (p<0.05, Fig. 4B). A reduction in RNF2 levels by siRNA knock-down, did not change the IC50 concentrations for gemcitabine (data not shown), which is consistent with the lack of altered RRM1 levels as shown earlier. However, the simultaneous depletion of both genes significantly (*p*<0.05; paired t-test) increased gemcitabine resistance (2.2-fold IC50 increase; Fig. 4C).

### Bmi1 and RRM1 *in situ* Protein Levels in Lung Cancers

To investigate the relationships between RRM1, RNF2, and Bmi1 in human tumor samples from patients with NSCLC, we used a tissue microarray consisting of 3 replicates of 187 surgically resected patients with stage I disease [19]. *In situ* protein levels of Bmi1 and RRM1 as determined by AQUA are summarized in table 1. We were unable to validate reagents for adequate determination of RNF2 levels. There was a very weak, but statistically significant, correlation between Bmi1 and RRM1 levels (r = 0.19, *p = *0.01); i.e., RRM1 levels tended to increase with increasing Bmi1 levels.

**Table 1:** Bmi1 and RRM1 *in situ* protein levels.

	Bmi1	RRM1
**All Cases**, (N = 187)		
Median	2,295	6,293
Range	179–8,241	2,304–14,770
**Tumor Histology,** Mean (N*)		
Adenocarcinoma	2,099 (96)	6,625 (88)
Squamous cell	2,628 (67)	5,990 (64)
Large cell	2,278 (22)	6,733 (21)
*p* – value	0.184	0.260
**Tumor Size,** Mean (N)		
≤2 cm	2,363 (55)	6,613 (54)
>2–≤3 cm	2,305 (43)	6,328 (43)
>3–≤5 cm	2,363 (55)	5,786 (50)
>5–≤7 cm	2,527 (18)	6,090 (17)
>7 cm	2,286 (9)	5,948 (9)
*p* – value	0.621	0.144
**Smoking Status,** Mean (N)		
Never	2,065 (11)	6,798 (11)
Former	2,294 (112)	6,597 (104)
Current	2,672 (48)	5,970 (46)
Unknown	1,937 (14)	6,798 (11)
*p* – value	0.482	0.051
**Gender,** Mean (N)		
Male	2,136 (100)	6,071 (91)
Female	2,552 (85)	6,551 (82)
*p* – value	0.183	0.493

*N, number of cases.

We found no significant (*p*>0.05) association between the *in situ* levels of Bmi1 and tumor type (adenocarcinoma, squamous cell carcinoma, and large cell carcinoma), tumor size (categorized as ≤2 cm, >2 to ≤3 cm, >3 to ≤5 cm, >5 to ≤7 cm, and >7 cm), smoking status (never smoker, if the patient had smoked less than 100 cigarettes during life time; former smoker, if the patient had quit for at least 1 year; and current smoker, for all others), and sex (Table 1).

## Discussion

Since RRM1 is a key determinant of gemcitabine efficacy [6], [7], [8], [10], understanding cellular processing of RRM1 may provide novel leads for optimizing its therapeutic benefit. Here, we report for the first time that the polycomb proteins RNF2 and Bmi1 function as the E3 ubiquitin ligase complex that regulates RRM1 ubiquitination and abundance with a commensurate impact on drug activity. RNF2 and Bmi1 are known to mediate ubiquitination of histone H2A, which impacts transcriptional activity. However, in contrast to our results, where RNF2 and Bmi1 both promoted RRM1 ubiquitination alone with at least additive activity when put together, Bmi1 did not have enzymatic activity by itself but was required to stimulate the H2A ubiquitin ligase activity of RNF2 [18]. Our results not only provide evidence for Bmi1 and RNF2 activities beyond those typically associated with polycomb function, but also raise the possibility that induction of RRM1 ubiquitination by its E3 ligases may enhance cellular sensitivity to gemcitabine.

We observed an apparent inverse relationship between RRM1 and Bmi1 levels in three gemcitabine resistant cell lines, while RNF2 levels appeared to be independent. While both, RNF2 and Bmi1, are targets for E3 ligase-mediated ubiquitination, only RNF2 has self-ubiquitinating activity [17]. The E3 ubiquitin ligase for Bmi1 is the CULLIN3/Speckle-type POZ protein complex [20]. This suggests that Bmi1 protein levels, rather than RNF2 levels, may be better suited as a biomarker to predict cellular response to gemcitabine. In an analysis of 187 tumor specimens from patients with early-stage, surgically resected disease and no prior treatment with any systemic agents including gemcitabine, we observed a weak but statistically significant positive correlation between RRM1 and Bmi1. Although seemingly contradictory to the *in vitro* observation, the results obtained from patients’ specimens should only be interpreted as demonstrating a dynamic range of expression across the spectrum of lung cancers without a clear association with tumor histology, size, or patient demographics. An evaluation of the interaction between expression levels of Bmi1 and RRM1 and gemcitabine efficacy requires availability and analysis of a dataset of patients that received gemcitabine therapy with prospectively collected clinical efficacy data. Analysis of such a dataset may reveal that a subgroup of patients exist in whom high Bmi1 levels are associated with low RRM1 levels and increased response to gemcitabine.

Bmi1 was the first known mammalian homologue of Drosophila polycomb proteins and was initially discovered through its ability to cooperate with c-Myc in the induction of lymphoma [21], [22]. Bmi1 has been shown to induce telomerase activity and immortalize human mammary epithelial cells through suppressing p16INK4A and p19ARF transcription resulting in diminished pRb and p53 function [23]. Bmi1 is also important in maintaining stem cell renewal in normal and malignant cells [24], [25], [26]. In addition to these oncogenic functions, Bmi1 overexpression has been implicated in resistance to platinum analogues, etoposide, and gemcitabine in selected *in vitro* models due to inhibition of apoptosis [27], [28], [29]. Since our data suggest an opposite role for Bmi1 in gemcitabine sensitivity; i.e., sensitization rather than resistance through the degradation of RRM1 as E3 ligase, it is possible that Bmi1’s role in drug activity is highly context dependent. This is supported by the lack of efficacy of bortezomib, a clinically approved proteasome inhibitor in multiple myeloma and mantle-cell lymphoma, in combination with gemcitabine or gemcitabine/carboplatin in patients with advanced solid tumors [30], [31].

In summary, our present work identifies RNF2 and Bmi1 as E3 ligases for RRM1 and that ubiquitination is a critical regulatory step in modulating RRM1 levels and cellular response to gemcitabine. This suggests that activating the ubiquitin pathway may be effective in treatment of gemcitabine-resistant lung cancer. Our data also suggest that Bmi1 levels, rather than RNF2 levels, may be better suited for assessment of gemcitabine efficacy in patients’ tumor specimens.

## Materials and Methods

### Cell Lines and Culture Conditions

Cell lines were maintained in RPMI 1640 or DMEM medium supplemented with 10% fetal bovine serum (FBS) and antibiotics. H23-Flag-RRM1 is a stable Flag-RRM1 overexpressing cell line, and H23-Ct is its corresponding control. Both were generated by transfection with full-length *RRM1* cDNA cloned into pCMV-Tag2 [5]. For cycloheximide treatment, the culture media were replaced with fresh DMEM without FBS. AD293, a derivative of the human embryonic kidney cell line HEK293 with improved adherence to culture dish surfaces, was obtained from Stratagene (catalog #240085).

### Reagents and Antibodies

Cycloheximide and MG132 were from Sigma, and gemcitabine was from Eli Lilly. The affinity-purified RRM1 antiserum R1AS-6 was generated in rabbits using an RRM1 peptide. Commercial Abs used included anti-RRM1 (Santa Cruz), anti-RNF2 (MBL International Corporation or Abcam), anti-Bmi1 (Upstate or Santa Cruz), anti-Ring1A (Cell Signaling), anti-ubiquityl-Histone H2A (Millipore), anti-Flag (Sigma), anti-HA (Sigma), anti-HSP60 (Cell Signaling), anti-GAPDH (Santa Cruz), anti-β-actin (Sigma), and anti-GFP (Santa Cruz).

### Plasmids and Transfections

RRM1 point mutations were created in pCMV-R1 [5]. HA-ubiquitin and mutants K63 and K48 were from Pablo Iglesias and Yixian Zheng (Johns Hopkins University). GFP-Bmi1 was from Maarten van Lohuizen. For co-precipitation analyses, 1×10^6^ cells were plated in 100-mm dishes in medium containing 10% FBS. One day after plating, cells were transfected with the indicated plasmids by Lipofectamine-2000 (Invitrogen). Cells were harvested after 48 h.

### Immunoprecipitation and Immunoblotting

Cell pellets were lysed in 50 mM Tris-HCl (pH7.4), 100 mM NaCl, 5 mM EDTA, 50 mM NaF, 10 mM NaPyrosphate, 200 µM Na3VO4, 1% Triton X-100, and a cocktail of protease inhibitors. Extracts were incubated with 1–3 µg of antibody coupled to protein G or A beads. IPs were eluted by boiling in SDS-PAGE loading buffer, electrophoretically separated, transferred to membranes, probed with the primary antibodies, and visualized with secondary antibodies using a chemiluminescent detection kit (Pierce). To detect RRM1 ubiquitination, cells were lysed with denaturing buffer (50 mM Tris [pH 7.5], 1% SDS, and 5 mM DTT) and heated at 95°C for 10 min. The lysates were then diluted to reduce SDS concentrations to 0.1% before the primary antibody was added.

### Immunofluorescence and Confocal Microscopy

Cells cultured on cover slips were fixed in 4% paraformaldehyde. They were permeablized with 1% glycin/0.5% Triton X-100, and blocked with PBS containing 10% FBS, 0.2% Triton-X100. Cells were then incubated with primary Ab in PBS containing 0.2% Triton X-100 and 1% bovine albumin followed by secondary Ab in PBS containing 1% bovine albumin. Slides were mounted with ProLong Gold antifade reagent containing DAPI (Invitrogen).

### *In vitro* Ubiquitination Assay

AD293 cells were transfected with Flag-RRM1, HA-RNF2, and GFP-Bmi1 and lysed after 48 h. Flag-RRM1 protein was immunoprecipitated with anti-Flag Ab cross-linked beads (M2-beads, Sigma), eluted by Flag-peptide (Sigma), and used as the substrate. HA-RNF2 and GFP-Bmi1 immunoprecipitated with anti-HA and anti-GFP Ab-conjugated agarose (Pierce) were used as E3 ligases. Flag-RRM1 (2.5 µL) and 20 µL anti-HA, anti-GFP IPs were incubated with 500 ng of E1 (Boston Biochem), 500 ng of E2 (UbcH5b and UbcH5c; Boston Biochem), and 5 µg His_6_-ubiquitin (Boston Biochem) in 50 µl reaction buffer (50 mM Tris [pH 7.5], 2.5 mM MgCl_2_, 15 mM KCl, 1 mM dithiothreitol, 0.01% Triton X-100, 1% glycerol, 8 mM ATP, 25 µM MG132, and protease inhibitors) at 37°C for 1 h. The reactions were terminated with SDS buffer containing β-mercaptoethanol and processed for 10% SDS-PAGE. Western blotting experiments were performed using the anti-ubiquitin antibody.

### Site-directed Mutagenesis

All primers were obtained from Integrated DNA Technologies. For K548R mutation, the forward and reverse primers were GCTGTGACCTTGCCAGGGAGCAGGG CCCATAC and GTATGGGCCCTGCTCCCTGGCA AGGTCACAGC. For K224R mutation, the primers were GTTTTCTTCTGAGTATGAGAGATGACAGCATTGAAGG and CCTTCAATGCTGT CATCTCTCATACTCAGAAGAAAAC. The QuikChange II site-directed mutagenesis kit (Agilent Technologies) was used to mutate A → G at positions 1802 and 830 in the human RRM1 cDNA respectively resulting in a K → R amino acid change respectively. K548/224R double-site mutations were generated using the K548R RRM1 as a template. Presence of the correct mutation was confirmed by sequencing.

### Target gene Expression Reduction

Dharmacon on-TARGETplus Smartpool siRNA to RNF2 and Bmi1 were delivered using Lipofectamine RNAiMAX (Invitrogen). Non-target Pool siRNA was used as control.

### Cell Viability Assay

Cell viability in response to gemcitabine was assessed with a tetrazolium-based cell proliferation assay (MTS). Briefly, 1,000–4,000 viable cells were seeded in 100 µL of growth medium in triplicate in 96-well plates. After 24 h, cells were transfected with siRNA for 24 h, and then exposed to gemcitabine at the desired concentration for 4 days. Cell viability was calculated using formazan absorbance. Each experiment was repeated 3 times on different days with separate preparations of cells and drug.

### Bmi1 and RRM1 *in situ* Protein Analysis

The tissue microarrays have been previously described [19] and consisted of three replicates from surgically resected and routinely processed non-small-cell lung cancers (NSCLC). Sections of 5 µm thickness on adhesive-coated glass slides were deparaffinized and processed for antigen retrieval. The reagents and dilutions used for target detection were D20B7 (rabbit monoclonal, Cell Signaling, #6964), 1∶25 for Bmi1 and R1-E4138-C42 (rabbit monoclonal, generated against full-length RRM1), 1∶1 for RRM1. Cytokeratin was used for identification of carcinomatous cells (murine anti-human pancytokeratin AE1/AE3, 1∶200, #M3515, Dako Cytomation). Slides were washed and incubated with two different secondary antibodies for 1 hr (Envision labeled polymer-HRP anti-rabbit, # K4011 and anti-mouse, # K4007, specific to the primary antibody used for target protein detection, 1∶200; Alexa 555 goat anti-mouse, #A21424, or goat anti-rabbit, #A21429, based on the source of the anti-cytokeratin, 1∶200, Dako Cytomation). For fluorescence amplification, slides were exposed to Cy5-Tyramide (1∶50) for 10 min at room temperature and mounted with Prolong Gold antifade reagent with DAPI. The final slides were scanned with SpotGrabber, image data were captured with a digital camera and fluorescence microscope, and *in situ* detection of targets and quantification of expression levels in the nuclear compartment was done using an immunofluorescence-based automated quantitative analysis (AQUA) system (PM-2000, version 2.3.4.1, HistoRx, New Haven, Connecticut) [32].

### Statistical Analysis

The average value of three tissue microarrays spot replicates was calculated for each patient. Associations among parameters with continuous values were calculated using the Spearman rank correlation coefficient. Associations between continuous and categorical values were calculated using the Wilcoxon rank-sum test for variables with two categories and the Kruskal-Wallis test for variables with three or more categories.
