# Expression of Concern: Ubiquitination and Degradation of Ribonucleotide Reductase M1 by the Polycomb Group Proteins RNF2 and Bmi1 and Cellular Response to Gemcitabine

**DOI:** 10.1371/journal.pone.0335988

**Published:** 2025-11-04

**Authors:** The PLOS One Editors

After this article [1] was published, concerns were raised regarding results presented in Fig 3 and Table 1.

Specifically:

- In Fig 3B lanes 1 and 2 appear similar
- In Table 1, the Bmi1 Tumor Size ≤2 cm and the Bmi1 Tumor Size >3-≤5 cm results show the same mean and number of cases.
- The published article [1] does not include information regarding institutional ethics approval for the collection of human samples.

The corresponding author stated that lanes 1–3 of Fig 3B are negative controls, that all lanes were run on the same gel, and that the original underlying blot data for Fig 3B are no longer available.

Regarding Table 1, the corresponding author stated the Bmi1 values for all tumor sizes are similar and are not significantly different. They provided summary data underlying Table 1 (S1 File), and stated that the individual-level data for Table 1 are no longer available. The *PLOS One* Editors noted that in S1 File, the Number of Samples for the Bmi1 121220 Nuclear AQUA v2.3. results is 185, whereas the sum of the number samples (N) for the Bmi1 levels for the Tumor Size results is 180 samples. The corresponding author stated that the total number of samples analyses was 185, but only 180 samples had both protein level values for Bmi1 and tumor size results available for analysis.

Regarding the ethics approval for the collection of human samples, the corresponding author stated the use of human tissue was approved by the Moffitt Cancer Center in Tampa, Florida with approval number MCC-13597 and the protocol included patient consent for use of residual tissue in a deidentified manner. They stated the AQUA analysis for RRM1 was performed at the Moffitt Cancer Center and the AQUA analysis for Bmi1 was performed at the Karmanos Cancer Institute (Wayne State University) with approval number 085510MP4X in reference to protocol number 2010−079. The *PLOS One* Editors requested further clarification on whether the patients provided written informed consent, but did not receive this information.

The *PLOS One* Editors issue this Expression of Concern to provide details of the ethics approval, and to inform readers of the above concerns with Fig 3 and Table 1 that cannot be fully resolved in the absence of the underlying data.

## Supporting information

S1 File Summary data underlying Table 1 Bmi 1 results. (XLSX)
